# Fungal Community Responses to Natural Humus Amendment Potentially Facilitate the Enhancement of Saline–Alkali Soil Multifunctionality

**DOI:** 10.3390/microorganisms13122877

**Published:** 2025-12-18

**Authors:** Xiaoting Sun, Jing Lei, Hang Chu, Yimin Liu, Fei Liu, Yang Li, Xuejia Zheng, Hui Zhang, Hui Pan, Congzhi Zhang, Qicong Wu

**Affiliations:** 1 China Rongtong Agricultural Development Group (Jinan) Corporation Limited, Jinan 250116, China; 13375681767@163.com (X.S.); athenalei@126.com (J.L.); 19937628222@163.com (H.C.); 2 China Rongtong Agricultural Development Group (Nanjing) Corporation Limited, Nanjing 210028, China; rtnfhr@126.com (Y.L.); masliufei@163.com (F.L.); 3 Co-Innovation Center for Soil-Water and Forest-Grass Ecological Conservation in Yellow River Basin of Shandong Higher Education Institutions, College of Forestry, Shandong Agricultural University, Tai’an 271018, China; 17543052998@163.com (Y.L.); zhengxuejia1212@163.com (X.Z.); 4 Institute of Food Safety and Nutrition, Jiangsu Academy of Agricultural Sciences, Nanjing 210014, China; 20100025@jaas.ac.cn; 5 State Experimental Station of Agro-Ecosystem in Fengqiu, State Key Laboratory of Soil and Sustainable Agriculture, Institute of Soil Science, Chinese Academy of Sciences, Nanjing 210008, China; 6 View Sino Orise Technology Limited, Jiangyin 214400, China; ph18@163.com

**Keywords:** natural humus, soil multifunctionality, soil microorganisms, microbial nutrient limitation, coastal saline-alkali land, fungal communities, soil amendment

## Abstract

Natural humus, characterized by its high organic carbon content and high degree of humification, is widely used in soil improvement. However, the impact of natural humus on the multifunctionality of saline–alkali soils and its relationship with soil microbial diversity remain poorly understood. This study conducted experiments with varying concentrations of natural humus to explore changes in soil multifunctionality and its driving factors. The results indicate that the addition of natural humus increases soil organic matter (by 23.5–45.73%) and alkali-hydrolyzable nitrogen (by 40–81.57%), while reducing electrical conductivity (by 1.8–35.9%). These changes enhance soil microbial diversity and improve soil multifunctionality. As natural humus is a high C/N material, nitrogen limitation in soil microorganisms may occur with increasing humus addition. However, the increase in K-strategy fungi (which are more efficient in resource utilization) helps maintain a relatively high level of soil multifunctionality. At the maximum application rate (30 t/ha), soil multifunctionality reached its peak value of 0.41. These findings highlight the significant role of natural humus in improving saline–alkali soils and enhancing soil functions, particularly through its effects on microbial communities, especially K-strategy fungi.

## 1. Introduction

Soil salinization is a significant global environmental issue, limiting food security and land use efficiency [1]. It disrupts critical ecological functions, such as soil structure, nutrient cycling, and water retention, leading to declining soil fertility and reduced agricultural productivity [2,3,4,5]. Traditional evaluations based solely on single functions, such as nutrient cycling, are insufficient to fully reflect the diverse ecological and productive capacities of saline–alkali soils. This limitation has led to the introduction of Soil Multifunctionality (SMF), a concept that assesses soil’s ability to deliver multiple ecological and productive functions simultaneously [6,7]. SMF offers a more holistic evaluation by considering various indicators, including soil nutrient status and physicochemical properties [8]. High levels of SMF are essential for maintaining agricultural productivity. Thus, enhancing soil fertility and understanding the mechanisms behind these changes are critical for the restoration and sustainable use of saline–alkali land.

Adding exogenous organic matter, particularly organic fertilizer, is an effective strategy for improving SMF [9]. Natural humic materials are formed over long periods from woody plants in moist, anaerobic environments [10]. As a stable exogenous organic material with a complex structure and a high carbon-to-nitrogen ratio, its elevated carbon content plays a significant role in improving soil fertility, particularly in enhancing soil organic matter [10,11]. However, this high-carbon characteristic also carries potential risks: if the carbon-to-nitrogen ratio of exogenous organic matter is excessively high, it may trigger strong microbial nitrogen immobilization during the initial decomposition stage, thereby leading to nitrogen limitation [12]. This nitrogen-limited state may weaken nitrogen cycling functions in the short term, thereby potentially exerting a negative impact on nutrient cycling within soil multifunctionality. Consequently, natural humic sub-stances might induce nitrogen limitation due to their relatively high carbon content. Therefore, natural humic substances may induce nitrogen limitation due to their relatively high carbon content. The soil microbial community plays a crucial role in regulating soil nitrogen cycling [13]. Soil microorganisms also secrete extracellular enzymes to meet their growth and metabolic needs [14]. Soil microorganisms also rely on diverse enzyme systems to acquire nutrients and maintain metabolic balance. Recent studies have highlight-ed the importance of microbial enzymatic pathways and metabolic interactions in sustaining ecological functions under stress conditions [15]. Moreover, recent studies have found that soil microorganisms play an important role in the degradation of pollutants [16]. Soil nutrient content has a significant impact on soil microbial diversity, which in turn plays a key role in maintaining soil function [17,18]. Currently, based on different life history strategies, soil microorganisms are commonly classified into two categories: r-strategists and K-strategists [19]. Generally, r-strategists are considered copiotrophic with a low resource affinity, while K-strategists are considered oligotrophic with a high resource affinity [20,21]. Studies have shown that under nitrogen-limited conditions with other resources being sufficient, oligotrophic microorganisms tend to dominate [22]. Therefore, classifying microorganisms based on their survival strategies helps in studying the functional roles of soil microorganisms under changing environmental conditions. Based on the above, this study aims to investigate the relationships among natural humic substance addition, SMF, and soil microbial communities.

The primary objective of this study is to evaluate the effects of natural humus addition concentrations on SMF and soil microbial communities, thereby elucidating the relationship between the two. Our hypotheses are as follows: (1) natural humus addition enhances SMF by improving the soil microenvironment and modifying microbial community structure; (2) as natural humus addition increases, microbial N limitation gradually rises, potentially reducing multifunctionality. In this study, typical medium to severe saline–alkali soil from coastal areas was collected and used to set up pot experiments to verify these two hypotheses. The research results will enhance the understanding of the complex interaction mechanism among natural humus, microorganisms, and SMF, providing theoretical support for the improvement and utilization of coastal saline soil.

## 2. Materials and Methods

### 2.1. Experimental Site, Design, and Sampling

This experiment focused on heavily saline–alkali soils from the coastal area of Weifang City, Shandong Province. The test soils had a pH exceeding 9, salinity exceeding 6 g/kg, and SOM below 10 g/kg. Soil samples were collected on 1 March 2024 and transported back to the laboratory. After air-drying at room temperature and removing stones and other impurities through a 2 mm sieve, corn pot experiments were conducted. The experiment was conducted from March to September 2024 at the Shandong Agricultural University Experimental Station. Plastic pots (26 cm in diameter) were used for planting, with each pot containing 5 kg of soil. The experiment included four different natural humus application rates: 0 t/ha natural humus (CK), 7.5 t/ha natural humus (NHL), 15 t/ha (NHM), and 30 t/ha natural humus (NHH). Each treatment had three replicates. To ensure consistency across treatment groups, all pots were initially placed randomly. Throughout the potting experiment, all specimens were maintained under uniform environmental conditions, including consistent light and temperature. Additionally, watering was conducted at fixed intervals using the same volume of water each time to stabilize soil moisture. The test crop was corn, which was sown on 14 March 2024. Three corn seeds were sown per pot. After seed germination and emergence, one plant per pot was retained. Prior to sowing, basal applications of N, phosphorus, and potassium fertilizers were applied to all treatments at rates of 225 kg/ha, 120 kg/ha, and 90 kg/ha, respectively. All treatments also received top-dressing with urea. Soil and plant samples were collected on 14 September 2024. The collected soil samples were divided into three portions for laboratory analysis: one portion was used for soil aggregate fractionation; another portion had roots, small stones, and other impurities removed, was air-dried naturally, and then passed through a 2 mm sieve for determining soil physicochemical properties; and the remaining portion was stored at −80 °C for assessing soil microbial indicators.

### 2.2. Determination of Soil and Plant Properties

Soil organic carbon (SOC) was determined using the potassium dichromate oxidation method [23]. The procedure involved mixing 0.2 g of soil sample with potassium dichromate (Tianjin Kaitong Chemical Reagent Co., Ltd., Tianjin, China) and sulfuric acid(Sinopharm Chemical Reagent Co., Ltd., Shanghai, China), followed by heating to ensure complete oxidation. Subsequently, the remaining potassium dichromate was titrated with ferrous sulfate (Tianjin Kaitong Chemical Reagent Co., Ltd., Tianjin, China). Finally, the organic carbon content was calculated based on the volume of ferrous sulfate consumed. Soil alkali-hydrolyzable nitrogen (AN) is determined using the alkali diffusion method, with the following procedure [23]: First, place 2 g of soil sample in the outer chamber of the diffusion vessel and add boric acid (Tianjin Kaitong Chemical Reagent Co., Ltd., Tianjin, China) absorption solution to the inner chamber. Then, rapidly add sodium hydroxide (Tianjin Kaitong Chemical Reagent Co., Ltd., Tianjin, China) solution to the opposite side of the outer chamber and immediately seal the vessel. After incubating at a constant temperature for 24 h, titrate the absorption solution in the inner chamber with a standard acid solution. Finally, calculate the AN content based on the volume of acid consumed during titration. Soil pH and electrical conductivity (EC) were determined using electrodes (Shanghai Yidian Scientific Instrument Co., Ltd., Shanghai, China) with a soil-to-water ratio of 2.5:1. Soil moisture content (SWC) was evaluated using the oven-drying method. Soil bulk density (SBD) and total porosity (TP) were both determined using the ring knife method [24,25]. Aggregate stability was determined using the dry sieve method [26]. In this method, a standard set of dry sieves was employed to classify air-dried soil samples into four distinct particle size fractions: aggregates and debris with diameters of <0.053 mm, 0.053–0.25 mm, 0.25–2 mm, and >2 mm. Using the aforementioned data, the mean weight diameter (MWD) and GMD were calculated via the following formula [27]:

(1) MWD = ∑i=1nXi×Wi∑i=1nWi

(2) GMD=exp∑i=1nWilnXi∑i=1nWi

where X_i_ and W_i_ are the average diameter and corresponding proportion of the soil aggregate grain size for two adjacent grain grades, respectively, and n is the number of soil aggregate sizes that were classified.

Above-ground biomass (AGB) of corn was determined by drying at 75 °C to constant weight [28]. Below-ground biomass (WR) was determined by thoroughly rinsing the root system, drying at 85 °C to constant weight, and weighing using a precision balance [29].

### 2.3. Quantification of Enzzyme Activities and Nutrient Limitation

We purchased the kit from Geruisi Biological Co., Ltd. (Suzhou, China) and performed the activity assays for β-glucanase (BG), N-acetylglucosaminidase (NAG), alkaline phosphatase (AP), and L-leucine aminopeptidase (LAP) according to the instructions provided in the kit. Enzyme stoichiometry was determined by calculating ratios between different enzyme activities. The first method involves calculating two ratios: BG/(LAP + NAG) and BG/AP. Higher values indicate reduced N and phosphorus limitations on microbial activity. The second method employs vector analysis to compute vector length (VL) and vector angle (VA), with the formulas defined as follows:

(3) $$VL\;=\;\sqrt{{(BG/\left[LAP\;+\;NAG\right])}^{2}}\;+\;{(BG/AP)}^{2}$$

(4) $$VA=Degrees(ATAN2\left(BG/AP\right),(BG/\left[LAP+NAG]\right)$$

A longer VL indicates a greater degree of C limitation on microorganisms; a VA less than 45° indicates N limitation; a VA greater than 45° indicates phosphorus limitation. The greater the deviation of VA from 45°, the stronger the N or phosphorus nutrient limitation on microorganisms [30].

### 2.4. Quantitative Calculation of Soil Multifunctionality

This study selected 13 indicators—SOM, AN, pH, EC, SBD, SWC, TP, AP, BG, LAP, NAG, mean weight diameter (MWD), and unstable aggregate index (SWA)—to calculate SMF. SMF was calculated using the mean value method proposed by Hooper and Vitousek (1998) [[31]. All soil indicators were first standardized using Z-scores, calculated as follows:

(5) $$Z_{ij\;}=\;(X_{ij}\;-\;u_{i})/\sigma_{j}$$

where Z*_ij_* denotes the Z-score for the jth soil functional indicator in the ith plot; *X_ij_* denotes the measured value of the jth soil functional indicator in the ith plot; *u_j_* represents the mean value of the jth soil functional indicator across all plots; and *σ_j_* indicates the standard deviation of the jth soil functional indicator across all plots.

SMF represents the average of all standardized soil functional indicator Z-scores within the plot, calculated as follows:

(6) $$SMF\;=\;\frac{1}{F}\sum_{i=1}^{F}g(f_{i})$$

where SMF denotes soil multifunctionality, F represents the number of soil functional parameters, f_i_ indicates the measured value of function i, and g is the standardization function.

### 2.5. Microbial Community Composition Revealed by Illumina Sequencing

Soil DNA was extracted and subjected to PCR amplification, targeting the bacterial 16S rRNA gene with primers 515F/806R and the fungal 18S rRNA gene with primers 528F/706R. The purified PCR products were sent to Novogene (Beijing, China) for paired-end sequencing on an Illumina platform. Subsequent bioinformatic analysis involved classifying the bacterial and fungal sequences at the phylum level. Based on taxonomic assignment, these phyla were further categorized as either K-strategists or r-strategists (Table A1).

### 2.6. Statistical Analyses

Data analysis was primarily conducted using SPSS version 27.0. Under the premise that the data satisfied the assumptions for parameter testing, a one-way analysis of variance (ANOVA) combined with Duncan’s multiple range test was employed to assess the significance of differences in soil physicochemical properties, soil nutrients, microbial data, and SMF among treatments. The significance level was set at *p* < 0.05. All figures were created and linear regression analyses were completed using Origin 2024.

## 3. Results

### 3.1. Soil Physicochemical Properties

**Figure 1:**
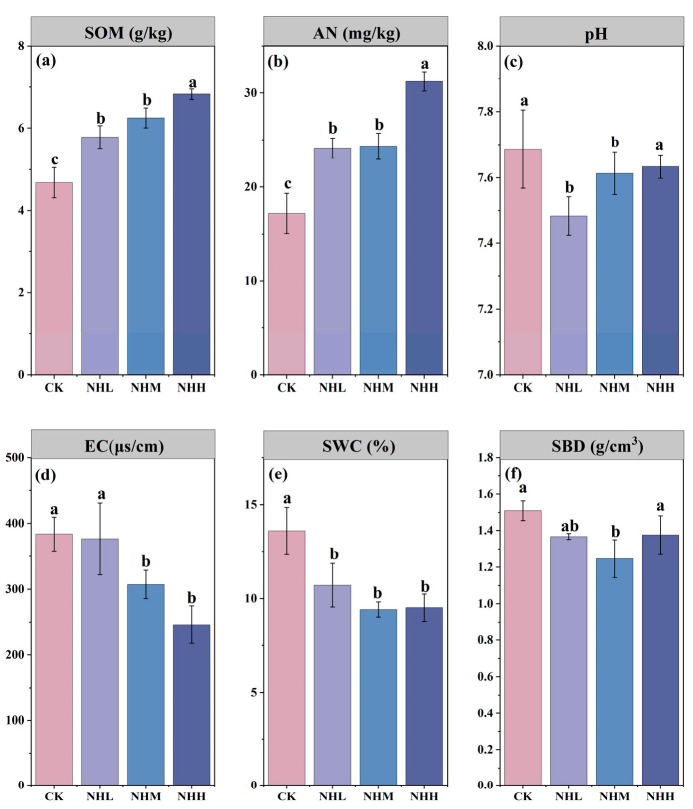
Soil physicochemical characteristics. (**a**) SOM: soil organic matter under different treatments; (**b**) AN: alkali-hydrolyzable nitrogen under different treatments; (**c**) pH under different treatments; (**d**) EC: electrical conductivity under different treatments; (**e**) SWC: soil moisture content under different treatments; (**f**) SBD: soil bulk density under different treatments. CK, NHL, NHM, and NHH indicate 0 t/ha natural humus, 7.5 t/ha natural humus, 15 t/ha, and 30 t/ha natural humus. Values are means ±  standard deviation (*n*  =  3). Lowercase letters show significant differences among treatments (*p* < 0.05, one-way ANOVA).

**Figure A1:**
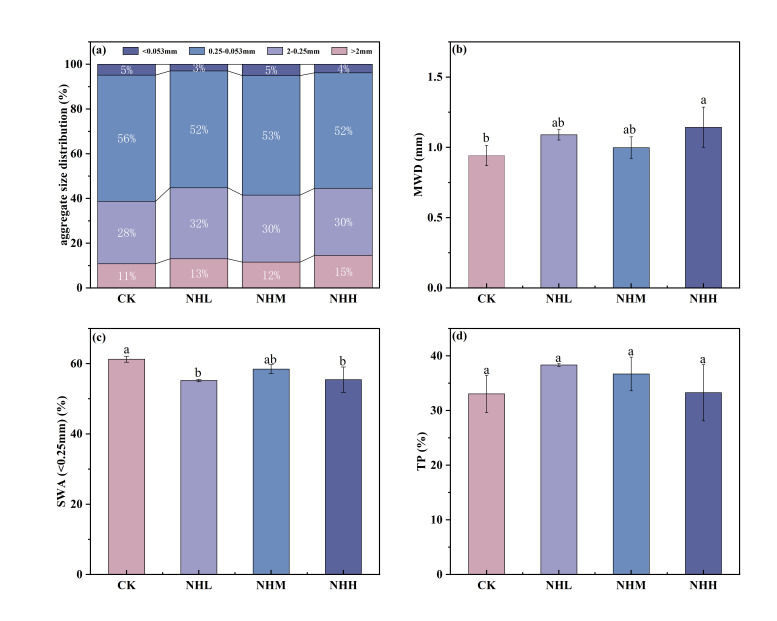
(**a**) Soil aggregate composition under different treatments; (**b**) MWD: mass median diameter under different treatments; (**c**) SWA: unstable aggregate index under different treatments; (**d**) TP: total porosity under different treatments. CK, NHL, NHM, and NHH indicate 0 t/ha natural humus, 7.5 t/ha natural humus, 15 t/ha, and 30 t/ha natural humus. Values are means ±  standard deviation (*n*  =  3). Lowercase letters show significant differences among treatments (*p* < 0.05, one-way ANOVA).

As shown in Figure 1, the addition of natural plant material has a positive effect on improving soil physicochemical properties. Under the NHH treatment with the highest application rate of natural humus, SOM and AN reached peak values of 6.82 g/kg and 31.23 mg/kg, respectively, representing significant increases of 45.73% and 81.57% compared with the CK. In contrast, EC was reduced by the natural humus amendments. The NHM and NHH treatments decreased EC by 19.89% and 35.62%, respectively, compared with CK. The addition of natural humus increased the MWD and content of aggregates > 0.25 mm, while its influence on TP was minimal (Figure A1).

### 3.2. Soil Microbial Community Composition and Life History Strategies

**Figure 2:**
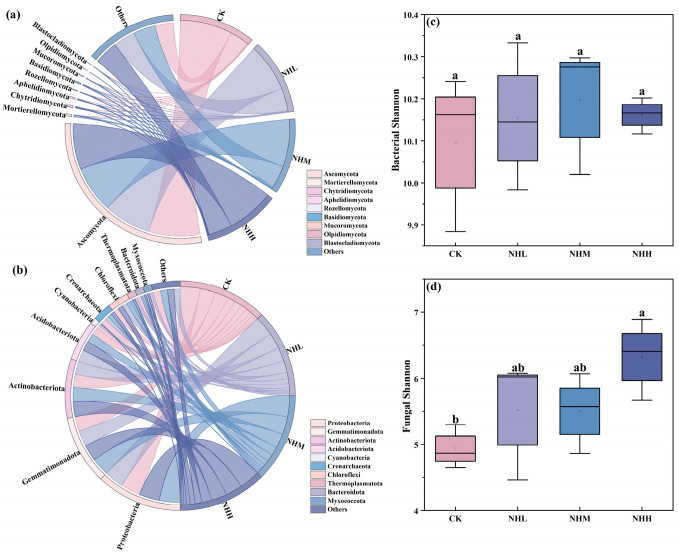
Microbial community structure and diversity. (**a**) Fungal phylum-level community composition chord diagram; (**b**) bacterial phylum-level community composition chord diagram; (**c**) box plot of the Shannon index for bacterial communities; (**d**) box plot of the Shannon index for fungal communities. CK, NHL, NHM, and NHH indicate 0 t/ha natural humus, 7.5 t/ha natural humus, 15 t/ha, and 30 t/ha natural humus. The box in a box and whisker plot represents the range of the mean plus or minus one standard error (SE). The whiskers indicate the outliers within 1.5 times the interquartile range (IQR). The line within the box denotes the median. Lowercase letters show significant differences among treatments (*p* < 0.05, one-way ANOVA).

The addition of natural humus did not significantly alter the bacterial Shannon’s index compared with the CK treatment (Figure 2c). In contrast, the fungal Shannon’s index increased significantly (*p* < 0.05), with the lowest Shannon’s index observed in the CK treatment at 4.94 (Figure 2d). At the phylum level, the bacterial communities were dominated by Proteobacteria, Gemmatimonadota, Actinobacteriota, and Acidobacteriota (Figure 2b), while the fungal communities were primarily composed of Ascomycota and Mortierellomycota (Figure 2a). To investigate the successional dynamics of microbial communities, we classified microbial taxa into r- and K-strategists based on their life history strategies. The results showed that following the addition of natural humus, there were no significant differences in the K/r ratio of bacteria, indicating that bacterial communities did not exhibit clear successional shifts. However, the K/r ratio of fungi showed a slight upward trend (Table 1). This trend suggests that fungal communities may be adapting to changes in soil properties, but the differences observed were not statistically significant. These findings highlight the subtle influence of natural humus on fungal community composition, which may require longer-term observation to fully understand its impact on microbial succession.

### 3.3. Soil Extracellular Enzyme Activities and Microbial Nutrient Limitation

**Figure 3:**
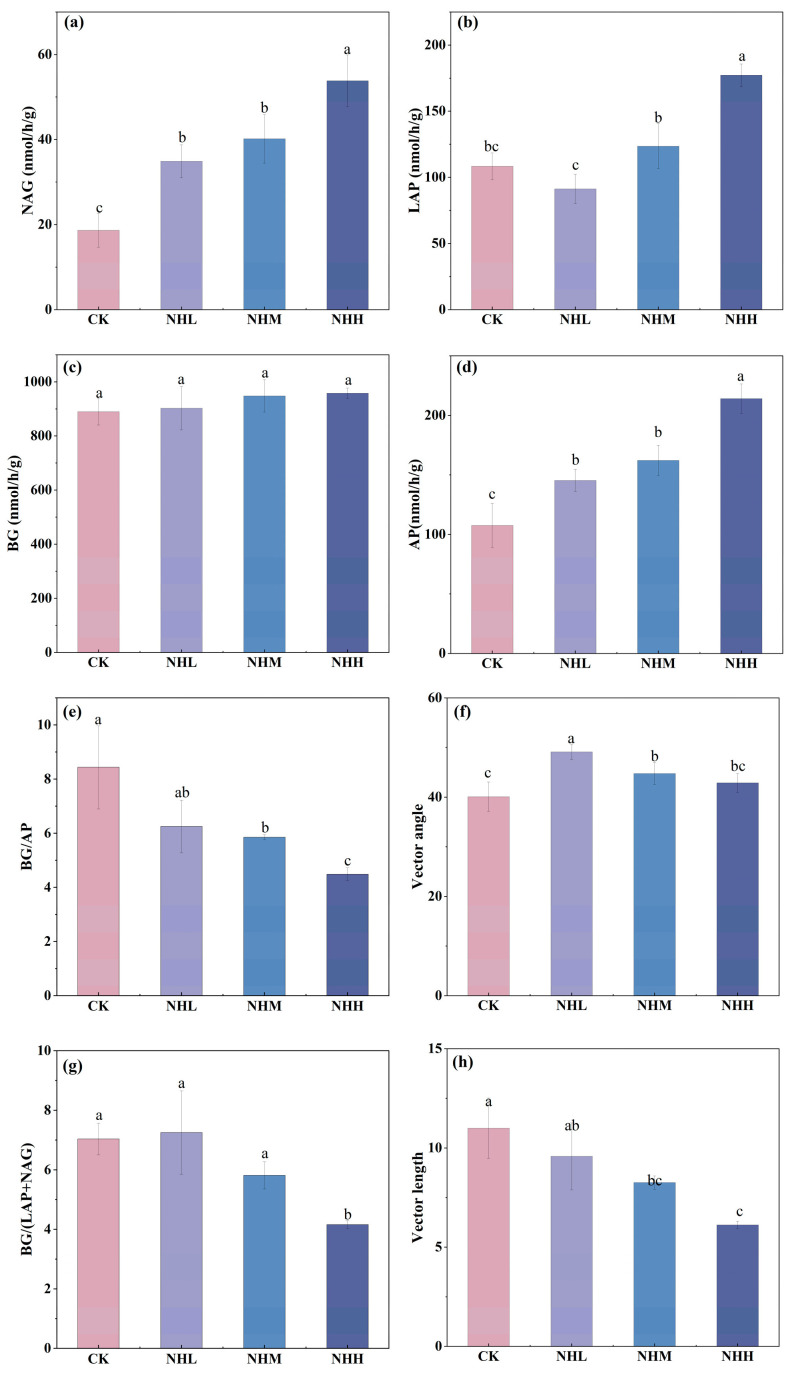
Soil enzyme activities and enzyme stoichiometry under different treatments. (**a**) NAG: N-acetyl-β-glucosaminidase under different treatments; (**b**) LAP: L-leucine aminopeptidase under different treatments; (**c**) NAG: N-acetyl-β-glucosaminidase under different treatments; (**d**) AP: alkaline phosphatase under different treatments; (**e**) BG/AP under different treatments; (**f**) VA: vector angle under different treatments; (**g**) BG/(LAP + NAG) under different treatments; (**h**) VL: vector length under different treatments. CK, NHL, NHM, and NHH indicate 0 t/ha natural humus, 7.5 t/ha natural humus, 15 t/ha, and 30 t/ha natural humus. Values are means ±  standard deviation (*n*  =  3). Lowercase letters show significant differences among treatments (*p* < 0.05, one-way ANOVA).

The application of natural humus significantly affected most of the measured soil enzyme activities (Figure 3a–d). Specifically, the activities of NAG, BG, and AP increased progressively with higher application rates, consistently following the order NHH > NHM > NHL > CK. Regarding NAG activity, NHL, NHM, and NHH treatments increased it by 87.18%, 115.56%, and 185.25%, respectively. AP activity increased by 35.18%, 50.83%, and 124.08% under NHL, NHM, and NHH treatments, respectively. Regarding BG activity, although NHL, NHM, and NHH treatments also elevated it to some extent, the effect of adding natural humus on BG activity did not reach a significant level.

Further analysis revealed that both BG/AP and BG/(LAP + NAG) showed a significant decreasing trend with increasing natural humus application rates (Figure 3e,g). Vector analysis indicated that VA decreased continuously with increasing application rates, dropping below the 45° threshold (Figure 3f). Following the application of natural humus, VL was significantly reduced, and VL progressively decreased with increasing application rates (*p* < 0.05; Figure 3h). The application of natural humus reduces soil C limitation, with these constraints diminishing progressively as application rates increase. In contrast, N limitation exhibited varying trends, with its constraints intensifying as the addition rate increased.

### 3.4. Soil Multifunctionality and Corn Biomass: Linkages and Key Drivers

**Figure 4:**
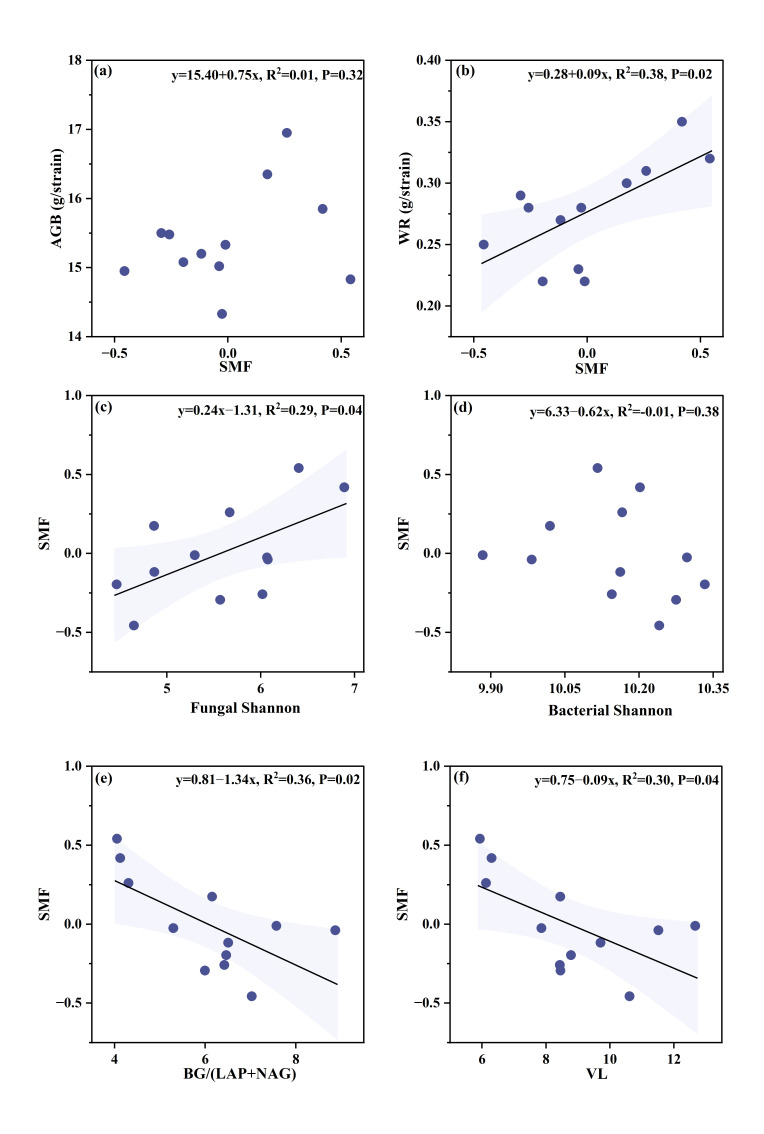
The relationship between SMF: soil multifunctionality and key indicators. (**a**) The relationship between SMF: soil multifunctionality and AGB: above-ground corn biomass; (**b**) the relationship between SMF: soil multifunctionality and WR: below-ground corn biomass; (**c**) the relationship between fungal Shannon’s diversity and SMF: soil multifunctionality; (**d**) the relationship between bacterial Shannon’s diversity and SMF: soil multifunctionality; (**e**) the relationship between BG/(LAP + NAH) and SMF: soil multifunctionality; (**f**) the relationship between VL: vector length and SMF: soil multifunctionality. Shaded areas are 95% confidence interval of the fitting.

**Figure 5:**
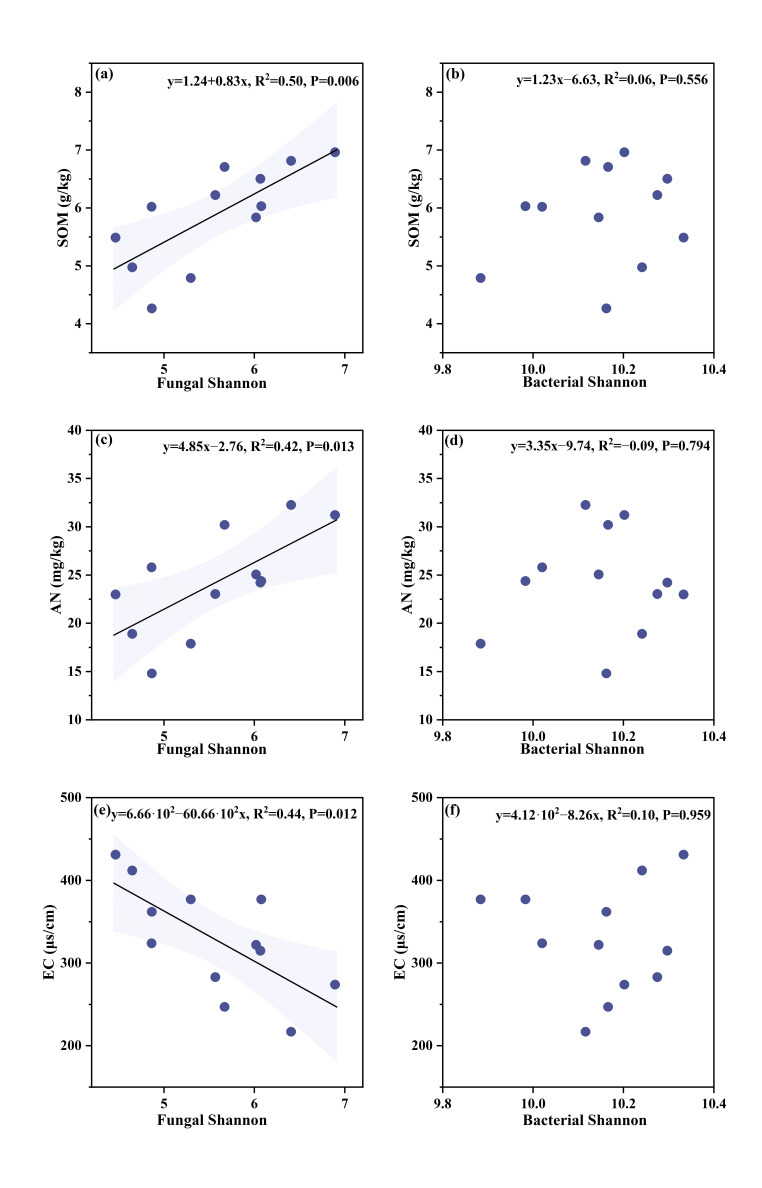
Relationship between fungal and bacterial Shannon’s diversity and soil physicochemical properties. (**a**) The relationship between fungal Shannon’s diversity and SOM: soil organic matter; (**b**) the relationship between bacterial Shannon’s diversity and SOM: soil organic matter; (**c**) the relationship between fungal Shannon’s diversity and AN: alkali-hydrolyzable nitrogen; (**d**) the relationship between bacterial Shannon’s diversity and AN: alkali-hydrolyzable nitrogen; (**e**) the relationship between fungal Shannon’s diversity and EC: electrical conductivity; (**f**) the relationship between bacterial Shannon’s diversity and EC: electrical conductivity. Shaded areas are 95% confidence interval of the fitting.

The addition of natural humus increased SMF, and the SMF continued to rise with the addition rate, reaching a maximum value of 0.41 in the NHH treatment (*p* < 0.05; Table 2). After adding natural humus, there was no significant change in the AGB of corn plants, while the AGB of NHH was 15.88 g/plant (Table 2). The underground biomass showed an upward trend with the addition of natural humus, where NHH exhibited the highest underground biomass at 0.33 g/plant (Table 2). Linear regression analysis revealed a significant positive correlation between SMF and underground biomass (*p* < 0.05; Figure 4b). Linear regression analyses revealed that the fungal Shannon’s index was significantly positively correlated with SOM and AN but negatively correlated with EC (*p* < 0.05; Figure 5a,c,e). Importantly, the fungal Shannon’s index was also positively correlated with SMF (*p* < 0.05), while no such relationship was observed for the bacterial Shannon’s index (Figure 4c,d). Furthermore, SMF was significantly negatively correlated with both the BG/(LAP + NAG) ratio and VL (*p* < 0.05; Figure 4e,f).

## 4. Discussion

### 4.1. The Addition of Natural Humus Increases SMF in Saline–Alkali Soils Primarily Through Fungal Communities

The results of this study support hypothesis 1, indicating that the addition of natural humus significantly enhances SMF in saline–alkali soils. The addition of humus in this study significantly increased soil organic matter and alkali-hydrolyzable nitrogen content, while reducing electrical conductivity (Figure 1). It also promoted an increase in fungal community diversity (Figure 2d) and ultimately enhanced soil multifunctionality (Table 2). This effect is primarily attributed to its improvement of the soil microbial environment. First, the results demonstrate that natural humus material, as a soil amendment, can im-prove environmental factors in saline–alkali soils when applied. Secondly, natural humus, as a material with a high organic matter content [11], significantly increases the organic matter content in the soil. Since soil carbon and nitrogen content is considered an important factor influencing soil fungal communities [32], the addition of natural humus in-creases the carbon and nitrogen content in the soil, making it a key factor in enhancing fungal diversity in this study. A study by Xu indicates that the addition of exogenous organic materials, such as compost, can improve soil quality by altering the fungal community structure in saline–alkaline soils [33]. Studies by Fan and Jiao also indicate that soil functionality is directly influenced by environmental factors and microbial communities [34,35]. In summary, our analysis suggests that the addition of natural humus improves soil physicochemical properties and enhances soil fungal community diversity. The combined effect of these factors ultimately increases SMF.

### 4.2. As the Amount of Added Natural Humus Increased, Microorganisms Gradually Became N Limited, Yet the Soil Microbial Community Maintained a High SMF by Adjusting Its Life History Strategies

Contrary to our initial assumption, the addition of natural humus did not inhibit multiple functions by exacerbating microbial N limitation. Instead, SMF significantly in-creased after being subjected to N limitation. This study indicates that as the application rate of natural humus increases, soil nitrogen limitation emerges and intensifies due to the high carbon-to-nitrogen (C:N) ratio of natural humus [11]. As the application rate rises, the overall C:N ratio of the soil input increases, reducing the nitrogen available to micro-organisms and leading to microbial nitrogen limitation [12]. Jiao et al. found that changes in soil moisture significantly affect microbial communities and respiration, especially under drought and wet conditions, further supporting the enhanced adaptability of microbial communities under nitrogen limitation [36]. In this study, we also observed an in-creasing trend in the K/r ratio within the fungal community (Table 1), reflecting a shift towards K-strategy microorganisms. This shift directly drives the remodeling of ecological functions, significantly enhancing the ability to decompose complex organic matter and the efficiency of nutrient mining [37]. K-strategy microorganisms act as efficient “pumps,” assimilating unstable carbon into their biomass during the decomposition of high C:N ratio organic matter, while releasing excess nitrogen [38]. Therefore, although microorganisms experience nitrogen limitation, their activity contributes to increased nitrogen flux and a more stable carbon pool, ultimately enhancing soil multifunctionality. Additionally, certain bacterial functional groups played a role. For example, the proportion of Proteobacteria in the bacterial community increased (Figure 3), as groups within Proteobacteria, such as denitrifying and nitrogen-fixing bacteria, can mitigate the effects of nitrogen limitation [39,40]. Despite strong nitrogen limitation at the individual microorganism level, nutrient cycling flux and carbon stability efficiency were synergistically enhanced at the community level, leading to an overall improvement in soil multifunctionality.

## 5. Conclusions

The incorporation of natural humus enhances SMF. Following its application, alterations in SOM, AN, and EC emerge as key factors influencing microbial community diversity. Shifts in microbial diversity, particularly among fungi, are closely linked to changes in SMF. With the increase in the application rate of natural humus, soil microorganisms exhibit a trend of intensified nitrogen limitation. However, by driving the microbial com-munity, particularly fungi, toward a K-strategy shift and promoting functional group synergy, the soil carbon and nitrogen cycling processes are reconstructed at a higher level, ultimately leading to enhanced soil multifunctionality. Although these findings provide important insights into the role of natural humus in improving soil functionality and re-claiming saline–alkali land, it should be noted that this study was conducted with a single soil type and limited environmental gradients, without encompassing the responses across different climatic regions and cropping systems. The specific mechanisms linking microbial changes to improvements in soil multifunctionality still require further validation through research under varied environmental conditions and over longer timeframes. Through rigorous experimental design and systematic multidimensional analysis, this study reveals a novel mechanism by which natural humus regulates soil functionality, offering an innovative bio-regulatory approach for reclaiming saline–alkali land, with significant practical implications.

## Figures and Tables

**Table 1:** The K/r ratio of fungi to bacteria in different treatments.

Group	Bacterial K/r	Fungal K/r
CK	2.026 ± 0.027 a	0.015 ± 0.003 a
NHL	2.261 ± 0.111 a	0.018 ± 0.006 a
NHM	2.016 ± 0.204 a	0.021 ± 0.004 a
NHH	2.093 ± 0.087 a	0.023 ± 0.005 a

Note: Different lowercase letters indicate significant differences between treatments (*p* < 0.05). Values represent mean ± standard error.

**Table 2:** Comparison of SMF: soil multifunctionality, AGB: above-ground corn biomass, and WR: below-ground corn biomass under different treatments.

Group	SMF	AGB (g/Strain)	WR (g/Strain)
CK	−0.19 ± 0.134 b	15.16 ± 0.112 a	0.25 ± 0.015 bc
NHL	−0.16 ± 0.093 b	15.19 ± 0.144 a	0.24 ± 0.019 c
NHM	−0.05 ± 0.192 b	15.39 ± 0.586 a	0.29 ± 0.006 ab
NHH	0.41 ± 0.114 a	15.88 ± 0.612 a	0.33 ± 0.012 a

Note: Different lowercase letters indicate significant differences between treatments (*p* < 0.05). Values represent mean ± standard error.

## Data Availability

The original contributions presented in the study are included in the article. Further inquiries can be directed to the corresponding authors.

## Abbreviations

The following abbreviations are used in this manuscript:

SMF	Soil multifunctionality
SOM	Soil organic matter
AN	Alkali-hydrolyzable nitrogen
EC	Electrical conductivity
SWC	Soil moisture content
SBD	Soil bulk density
AGB	Above-ground corn biomass
WR	Below-ground corn biomass
BG	β-glucosidase
LAP	L-leucine aminopeptidase
AP	Alkaline phosphatase
NAG	N-acetyl-β-glucosaminidase
VA	Vector angle
VL	Vector length
NHL	7.5 t/ha natural humus
NHM	15 t/ha natural humus
NHH	30 t/ha natural humus
CK	0 t/ha natural humus
MWD	Mass median diameter
SWA	Unstable aggregate index
TP	Total porosity

## Appendix A

**Table A1:** r-K selection bacterial/fungal phyla defined in the study.

	Phylum (Subphylum)	r-K Selection	References
Bacteria	Proteobacteria	r-selection	[41,42,43]
	Gemmatimonadota	K-selection	[44,45,46]
	Actinobacteriota	K-selection	[46,47,48]
	Acidobacteriota	K-selection	[19,41,49,50,51]
	Cyanobacteria	K-selection	[52]
	Crenarchaeota	K-selection	[53]
	Chloroflexi	K-selection	[46,47,48]
	Bacteroidota	r-selection	[46,48]
	Myxococcota	r-selection	[54]
Fungi	Ascomycota	r-selection	[48,55,56,57]
	Mortierellomycota	r-selection	[58]
	Chytridiomycota	r-selection	[52]
	Aphelidiomycota	K-selection	[52]
	Rozellomycota	K-selection	[55,56,57]
	Basidiomycota	K-selection	[53]
	Mucoromycota	K-selection	[53]

r-K selection-associated bacterial/fungal phyla defined in the study.
